# Case Reports of Cow's Milk Protein Allergy Presenting as Delayed Passage of Meconium With Early Onset Infant Constipation

**DOI:** 10.3389/fped.2022.858476

**Published:** 2022-04-15

**Authors:** Akshita Madala, Allison C. Lure, Svea Cheng, Sam X. Cheng

**Affiliations:** ^1^Department of Pediatrics, University of Florida, Gainesville, FL, United States; ^2^Department of Pediatrics, Gastroenterology, Hepatology, and Nutrition, University of Florida, Gainesville, FL, United States

**Keywords:** cow's milk protein allergy, cow's milk protein sensitivity, milk allergy, delayed meconium passage, early onset infant constipation

## Abstract

A cellular proliferation to milk allergens has been found in the cord blood cells of neonates. While this reflects a sensitivity during the fetal life, its clinical significance and disease, particularly its unconventional presentations, have remained largely unrecognized by care providers. Here, we report three cases of infants whose mothers consumed dairy products during pregnancy, who developed a severely constipated pre- and postnatal bowel. The passage of meconium was significantly delayed with subsequent early-onset infant constipation that was intractable to conventional therapies but remitted when milk proteins were withheld, recurred when milk proteins were reintroduced, and resolved again when switched to an extensively hydrolyzed or amino acid-based infant formula. Based on this and other observations, it is believed that these infants must have initiated and/or developed cow's milk protein allergy prenatally during fetal life. We suggest that a 2-week trial of cow's milk protein avoidance be applied to these neonate infants with early-onset constipation before an unnecessary invasive work-up for Hirschsprung disease and others is initiated per the current guidelines.

## Introduction

Cow's milk protein allergy (CMPA) is an abnormal immunological response(s) to cow's milk protein(s) [CMP(s)] that commonly occurs in infants and young children (1). Since no single laboratory test is diagnostic, the diagnosis still has to be clinical, based on indirect milk elimination and provocation procedures, and a care provider's awareness becomes extremely important in making it diagnosed. Before 1950, CMPA was rarely diagnosed. Since 1970, this condition has been well documented. Now, it is estimated to occur in 1.8–17% of formula-fed infants and 0.5% breastfed infants (2, 3). For CMPA to occur, an individual must be first exposed and subsequently develop an immunological response to cow's milk allergen, and this process will take weeks or months to occur. As a result, CMPA most commonly occurs within weeks or months after birth. Importantly, cow's milk allergens are not only transported orally and through a mother's breastmilk into infants' circulation and induce CMPA; earlier studies suggest that they can also pass through the placenta and amniotic fluid to sensitize fetuses (4, 5) and cause allergy (6–9). For example, the proliferative responses of cord blood lymphocytes to cow's milk allergens such as α-lactalbumin, ß-lactoglobulin, and α-casein were found in full-term neonates (7–9). This means that CMPA can also happen in neonate infants even before their first postnatal feeds occur. However, while the former is now well known to care providers, the latter is often overlooked, particularly when this early-onset CMPA presents with atypical symptoms in the absence of hematochezia as in the vignettes of this communication. In the latter case, most pediatricians including pediatric gastroenterologists neither recognize nor are they even aware of it. As a result, many atypical cases of neonate infants with early-onset CMPA were either left undiagnosed, resulting in a delay in initiating the appropriate treatment for this very treatable condition, or misdiagnosed with other diseases, leading to many unnecessary workups. Since this early-onset CMPA is not uncommon, in order to raise an awareness and improve the clinical care of this unique clinical entity, we feel that it is necessary to report it again to pediatric communities.

In this communication, we report three cases of infants whose mothers persistently consumed dairy products during pregnancy, who then developed a severely constipated bowel, both pre- and postnatally. Their passages of meconium and stools were significantly delayed. They were so severely constipated that it was intractable to conventional laxative therapies. Interestingly, when milk-based formula or breastmilk was withheld from them, constipation resolved; when milk was reintroduced to them, constipation recurred; when their diet was switched to an extensively hydrolyzed or an amino acid-based infant formula, constipation resolved again. Based on this, we diagnose that these infants have CMPA that developed through prenatal exposure and is now evident through perinatal life.

## Case Description and Diagnostic Assessment

### Case 1

A 6-week-old boy born at the gestation age of 29 weeks via a cesarean section due to maternal preeclampsia and breech presentation presents with intractable constipation and a delayed passage of meconium for 7 days immediately after birth until he was given a rectal suppository. After birth, he tried both breast- and bottle feeding without the relief of symptoms, but when he was nourished exclusively via parental nutrition without enteral feeds, his constipation problem got resolved with spontaneous bowel movements. He was discharged after 40 days of neonatal intensive care unit (NICU) stay. He was breastfed again and then switched to a milk-based infant formula. On this regimen, he had pellets of hard stools again, which required him to undergo rectal stimulation or use suppositories two times a day, still resulting in infrequent stools. He also had reflux and spit-up or vomiting up to eight times a day, after each feed. He has had poor weight gain, only gaining on average 1 g/day in the past 6–7 weeks. His physical exam was reassuring. His mother had Crohn's disease, while his father had a chronic constipation of unknown etiology. There was no family history of cystic fibrosis, Hashimoto's thyroiditis, or Hirschsprung disease. His mother consumed dairy products during pregnancy and while breastfeeding.

Due to the concern for CMPA, the infant's diet was changed from a milk based-infant formula to an amino acid-based infant formula, due to his mother's unwillingness to breastfeed. Symptoms resolved within 48 h after he switched to an amino acid-based infant formula. On this new regimen, he has had spontaneous bowel movements without the need for rectal stimulation or the use of suppositories, his vomiting after feeds has been reduced from eight times a day to two times a day, and over the past 2 weeks, his weight gain has increased and is now appropriate at 18 g/day.

### Case 2

A 3-month-old boy born at term presents with persistent constipation since birth and a delayed passage of meconium until 6 days of age. He has had hard, painful stools that result in screaming and crying and infrequent bowel movements, once every 4–5 days, and has been admitted twice for fecal disimpaction. In addition, he has had poor weight gain with frequent vomiting that occurs after every feed. His physical exam was reassuring. His family history revealed no cystic fibrosis, celiac disease, thyroid disease, or chronic constipation nor allergy, although his mother persistently consumed dairy products during pregnancy. At birth, he initially started on a milk-based formula, and since then, he has tried multiple milk-based formulas with no success in relieving constipation or other symptoms.

Suspecting CMPA, the infant's feeds were changed to an extensively hydrolyzed infant formula, given that his mother was not interested in breastfeeding. Since starting exclusively on the hydrolyzed formula, the patient had a significant improvement in symptoms. He now has approximately one soft stool a day without the need for external assistance. His vomiting improved from multiple episodes a day to only four times a week, and he is now appropriately gaining weight.

### Case 3

A 6-week-old girl born at the gestation age of 39 weeks and 2 days *via* vaginal delivery presents with early-onset, intractable constipation. At day 1 of life, she passed a smear of meconium, but immediately after birth, she presented with constipation with hard stools and infrequent bowel movements requiring rectal suppositories. She began to be breastfed at her first day of life and a milk-based formula supplement was added at her second day of life. After passing stool for the first 3 days, she did not pass stool in the following 4 days. She again did not pass stool for 10 days. Her formula was then changed from a milk-based formula to an extensively hydrolyzed infant formula at day 16 of life. Her physical exam was reassuring. Her workup including a rectal biopsy showed normal nerve innervation, thus ruling out Hirschsprung's disease. Due to experiencing continued constipation, her formula was switched again to a partially hydrolyzed one with added prune juice and her symptoms remained unchanged. Her feedings consisted of 30% breastmilk and 70% formula. Her mother consumed a considerable number of dairy products including cheese, yogurt, and soy milk and reported a history of chronic constipation. In addition, the patient has one sibling with a history of infant constipation and suspected CMPA.

Suspecting CMPA, the patient's feeds were changed to an amino acid based-infant formula, and her mother was counseled to either avoid breastfeeding or avoid dietary dairy products, and her mother chose the former. Constipation resolved within 72 h after the patient fed exclusively on the amino acid-based formula and avoiding breastfeeding. Six weeks later, she had a desire to go back to regular feeding. Symptoms recurred after 2 days of trial, but they remitted again 2 days after she was back on the amino acid-based formula. Right now, she continues on the amino acid-based formula and remains asymptomatic.

## Discussion

**Figure 1:**
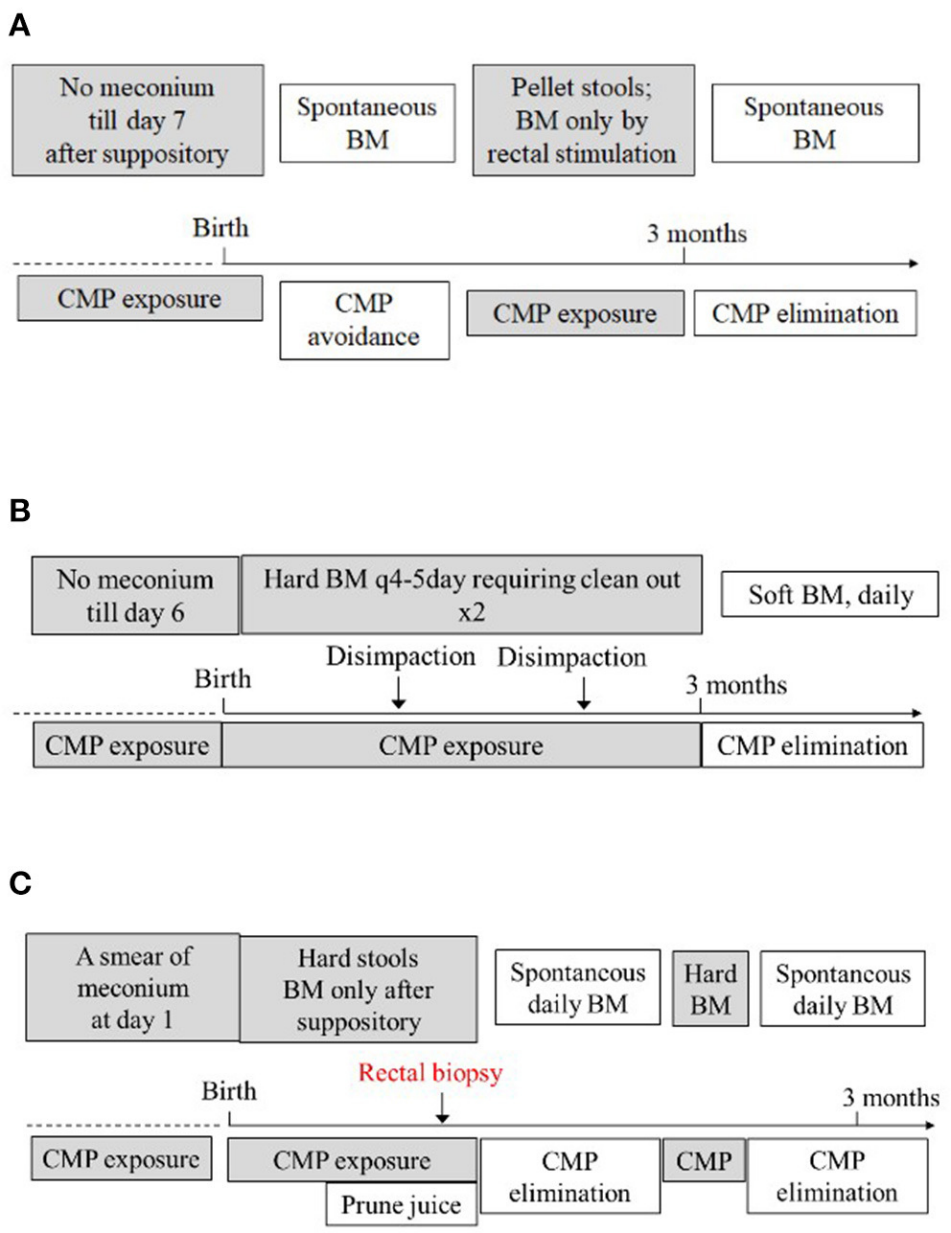
Summarizes the relationship between the exposure and elimination of CMP and expression of constipation in case 1 **(A)**, case 2 **(B)**, and case 3 **(C)**, establishing the diagnosis of CMPA in these patients. Refer to the text for detailed explanation. BM, bowel movement.

We present three unconventional cases of CMPA that presented at birth with a delayed passage of meconium/stool. Table 1 summarizes the main demographics and clinical symptoms of the three cases. Figure 1 summarizes the relationship between the exposure/elimination of CMP and the expression/resolution of constipation in case 1 (Figure 1A), case 2 (Figure 1B), and case 3 (Figure 1C), establishing the diagnosis of CMPA in these patients. In these three cases, the elimination of CMP was done through an extensively hydrolyzed or amino acid-based formula; however an alternative would be to eliminate CMP from maternal diet and thus from the breastmilk, allowing families to continue to breastfeed. In our cases, the families chose not to pursue this option. The infants' delayed passage of meconium/stool is not simply due to hard stool as the problem had remained unsolved when stool softeners were applied. Instead, their primary pathophysiology seems to be unable to move their bowel as in those with Hirschsprung disease. None of them had spontaneous bowel movement, and all required rectal stimulation for bowel movement. The facts that these infants did not respond to regular formulas but responded to an amino acid-based formula (cases 1 and 3), extensively hydrolyzed formula (case 2), or milk avoidance (case 1) suggest that the culprit is not the fats, carbohydrates, or other non-protein components in formulas but proteins. Indeed, symptoms remitted when CMPs were withheld (Case 1), recurred when CMPs were reintroduced (cases 1 and 3), and resolved again when CMPs were eliminated (cases 1–3), thus establishing the relationship between CMP exposure and the development of the disease.

**Table 1:** A summary of the age demographics and systemic and gastrointestinal symptoms of the three cases.

	**Post-menstrual age at birth**	**Age at presentation**	**Systemic symptoms**	**Gastrointestinal symptoms**
Case 1	29 weeks	Birth	Poor weight gain	Infrequent bowel movements, delayed passage of meconium, hard stool reflux, spit-up, vomiting
Case 2	Term	Birth	Poor weight gain	Infrequent bowel movements, delayed passage of meconium, hard stools, painful stools
Case 3	Term	4 days of life	None	Infrequent bowel movements, hard stools

Although our cases have established an association between CMP exposure and the development of slow-transit enteropathy, the mechanism of this association is unclear. Clinically, CMP can cause a slow-transit bowel at least in two ways: (1) nonspecifically through CMP constipation and (2) specifically through CMPA. CMPA, rather than CMP constipation, was diagnosed because for the latter to occur, patients require a prolonged consumption of large amounts of CMP similar to the changes in stool consistency caused by other dietary alterations, and this is unlikely to occur in our patients, neither *in utero* nor at day one of their lives. In addition, for the latter (constipation) to resolve, patients require the withdrawal of CMP for a prolonged period of time of at least 2 weeks, not 2 or 3 days as in our cases. Finally, infants with CMPA will usually also experience symptoms other than just constipation. In keeping with this, our patients had vomiting and a failure to thrive besides constipation, all resolved upon milk protein elimination, consistent with the diagnosis of CMPA.

For infants with a delayed passage of meconium and stool in the newborn period, we must consider Hirschsprung disease, the most frequently encountered congenital disorder of intestinal motility. However, the presence of rectal ganglion cells and resolution of the symptom in response to CMP elimination in our cases clearly speak against the diagnosis of this disorder. Other differential diagnoses include anorectal dyschezia, particularly the acquired form. However, infants with dyschezia will often have the 5–10 min period of fussing and red-contorted face straining prior to passing stool. They do so because their efforts to pass stool are uncoordinated due to a defective reflex for defecation. In addition, infants with dyschezia do not present with a delayed passage of meconium; they will not have gone 1 week without passing stool; they do not have to use a glycerin suppository or rectal stimulation to pass stool. Finally, infants with dyschezia should not respond to milk protein elimination.

Multiple mechanisms for CMPA have been suggested (3, 10–12). These include an immunoglobulin E (IgE)-mediated immediate hypersensitivity reaction through the mast cell release of histamine and a non-IgE-mediated delayed hypersensitivity reaction. The latter likely involves cytokine release from antigen-mediated T-cell stimulation or antigen-mediated immunoglobulin A (IgA) or immunoglobulin G (IgG) immune complex stimulation. Although the nature of reaction to CMPs in our patients is unknown, their symptoms and normal-appearing colorectal histology (case 3; not shown) did not suggest an IgE-mediated reaction. Therefore, the laboratory tests measuring CMP-specific IgE and skin test wheals are of limited diagnostic values and were not used in this study. In our rectal biopsy of the case 3 patient, no lamina propria/muscularis mucosa eosinophilia, lymphoid nodular hyperplasia, cryptitis, neutrophilic crypt abscess, glandular distortion, or Paneth cell metaplasia nor increased mast cell infiltration was observed. A more likely explanation would be a non-IgE-mediated mechanism.

**Figure 2:**
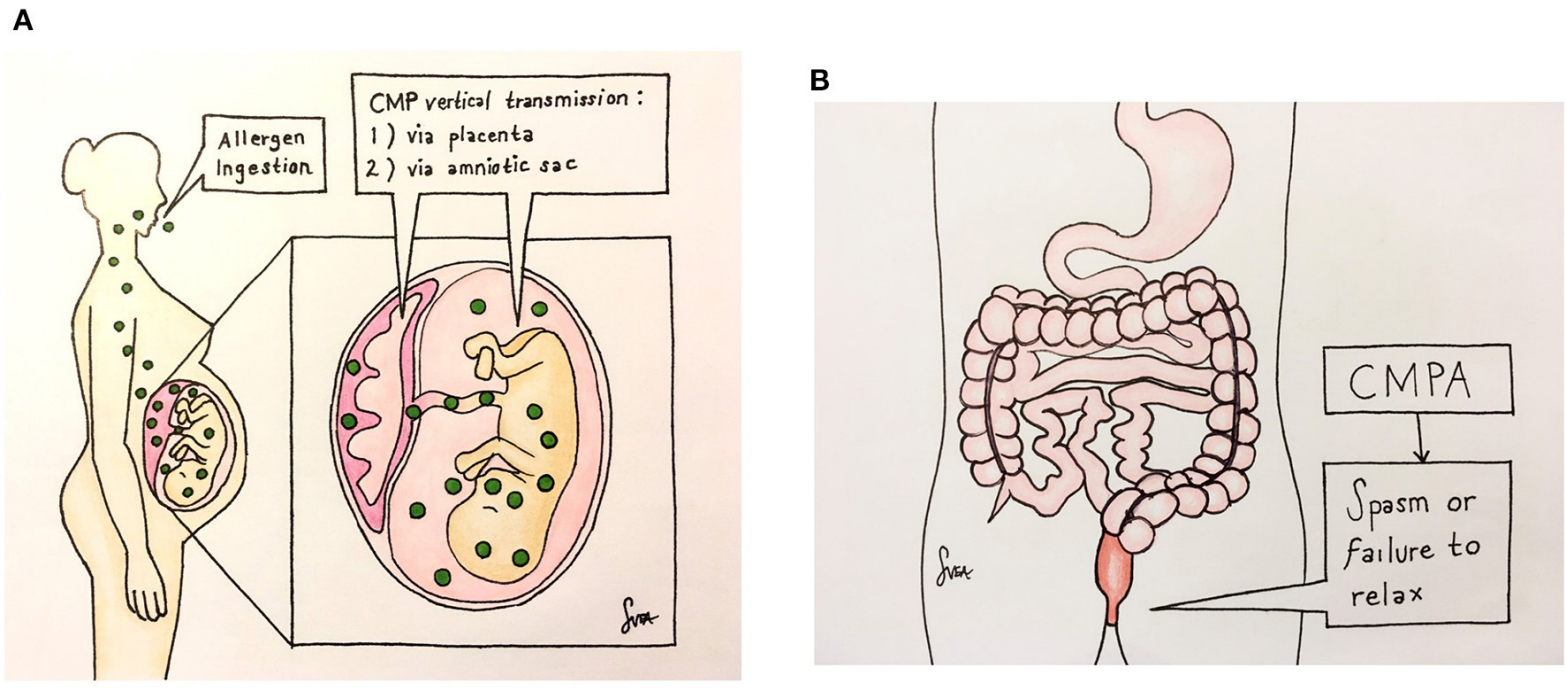
Illustrates how the maternal CMP exposure leads to the development of neonatal constipation. The CMP allergen is vertically transmitted during pregnancy from a mother to a fetus via the placenta and amniotic fluid to cause CMPA **(A)**. When the CMPA involves the colon, constipation results **(B)**. There, the allergy causes neuromuscular dysfunction by a yet-unknown non-IgE mechanism, leading to anal spasm or failure of the anal sphincter to relax, resulting in a functional obstruction.

Figure 2 illustrates how the maternal CMP exposure leads to the development of neonatal constipation. Cow's milk allergen is vertically transmitted during pregnancy from a mother to a fetus *via* the placenta and amniotic fluid to cause CMPA (Figure 2A). When the CMPA involves the colon, constipation results (Figure 2B). Although a detailed pathway(s) leading allergy to constipation is yet to be understood, it appears that the allergenic response involves neuromodulation of the enteric nervous system (ENS) (13), resulting in increased resting anal sphincter pressure and causing difficulties in defecation (14). In keeping with this, our three cases presented in this study all required anorectal maneuvers (suppository enema/thermometer insertion) to probably relieve anal pressure in order to defecate. Also, chronic functional constipation, once thought idiopathic, has now been regarded as neuropathologic due to a complex system of abnormalities of the ENS of these patients (15), and perhaps CMPA is the cause, at least in part, of such abnormalities.

CMP allergens can be transported orally and through a mother's breastmilk into infants' circulation and induce CMPA in infants. This is well established. Research also suggests that cow's milk allergens can pass through the placenta and amniotic fluid (7) to sensitize fetuses (16, 17), including fetal gut (16), at as early as 22 weeks of gestation (18) and cause CMPA. The latter includes conventional allergic colitis (19) and unconventional constipation (present study). This is important. This means that pediatricians must consider CMPA even in 1-day-old neonate infants as opposed to the currently taught manifestation at a few weeks or months of age. Were these neonates with early- onset CMPA left unidentified or untreated, they will become a more chronic clinical condition during childhood or adulthood. There are reports that up to 78% of chronic functional constipation later in life are probably CMPA-related (14).

The limitations of this report must be considered. The first is that sensitization and onset *in utero* were not shown directly. Second, CMPA was diagnosed clinically, rather than by laboratory tests such as an allergen-specific lymphocyte stimulation test (20) or prolonged, two-stage double-blind, placebo-controlled food challenges (18). In the future, we could consider performing cord blood cow's milk sensitization and/or allergen-specific lymphocyte stimulation tests in order to establish their correlation with the subsequent development of allergic disease. These tests, like other food allergy panels (e.g., skin prick tests and specific IgE tests), have a high negative predictive value; however, a positive result only indicates sensitization but not necessarily an allergy; in order to diagnose food allergy, the most appropriate testing remains clinically using an oral food challenge and elimination diet as in these vignettes (21, 22).

**Figure 3:**
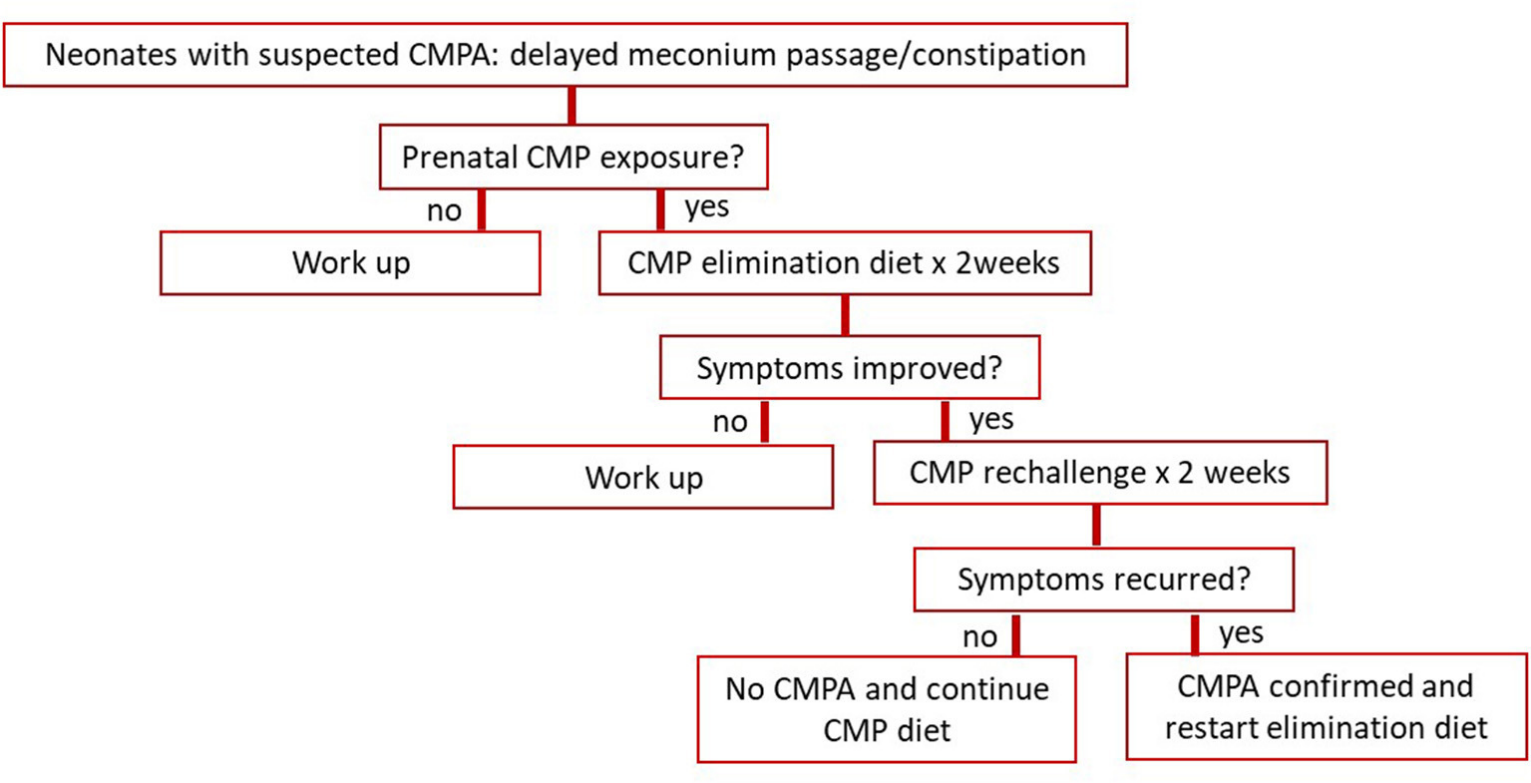
Proposes a management algorithm for early-onset constipation in neonates with suspected CMPA. CMP, cow's milk protein.

Given the discovery of the CMP hypersensitivity *in utero* and recognition of CMPA at birth, pediatricians should consider this condition in their differential diagnoses, particularly in newborns with a delayed passage of meconium and/or early-onset constipation suspicious of Hirschsprung disease or infant dyschezia, both congenital and acquired. Figure 3 proposes a new management algorithm for early-onset constipation in neonates with suspected CMPA. Considering its wide morbidity and simplicity in the treatment, we suggest that a 2-week trial of CMP avoidance be applied to these neonates and infants first. This way, an unnecessary invasive workup for Hirschsprung disease and others can perhaps be avoided.

## Data Availability Statement

The original contributions presented in the study are included in the article/supplementary materials, further inquiries can be directed to the corresponding author.

## Ethics Statement

Written informed consent was obtained from the minor(s)' legal guardian/next of kin for the publication of any potentially identifiable images or data included in this article.

## Author Contributions

SXC: conceptualized and designed the work. AM, AL, and SXC: collected and analyzed the data and interpreted the data. AM and AL: drafted the manuscript. AL, SC, and SXC: revised the manuscript. SXC: finalized this manuscript. The artwork of the Figures 2A,B were hand created by SC, and all authors approved the final version and agreed to be accountable for the content of this work.

## Conflict of Interest

The authors declare that the research was conducted in the absence of any commercial or financial relationships that could be construed as a potential conflict of interest.

## Publisher's Note

All claims expressed in this article are solely those of the authors and do not necessarily represent those of their affiliated organizations, or those of the publisher, the editors and the reviewers. Any product that may be evaluated in this article, or claim that may be made by its manufacturer, is not guaranteed or endorsed by the publisher.
